# Brain-to-brain synchrony during dyadic action co-representation under acute stress: evidence from fNIRS-based hyperscanning

**DOI:** 10.3389/fpsyg.2023.1251533

**Published:** 2023-09-06

**Authors:** Suqin Lin, Hanxuan Zhao, Haijun Duan

**Affiliations:** ^1^School of Preschool Education, Xi’an University, Xi’an, China; ^2^School of Business and Management, Shanghai International Studies University, Shanghai, China; ^3^Key Laboratory of Modern Teaching Technology, Ministry of Education, Shaanxi Normal University, Xi’an, China

**Keywords:** acute stress, joint Simon task, fNIRS, hyperscanning, action co-representation

## Abstract

Unexpected acute stressors may affect our co-representation with other co-actors when completing the joint tasks. The present study adopted the emergent functional near-infrared spectroscopy (fNIRS)-based hyperscanning method to explore the brain-to-brain synchrony when implementing the Joint Simon Task under acute stress induced in the laboratory. The behavioral results reported that the joint Simon effect (JSE) was found in both the stress group and the control group, but the joint Simon effect in the stress group was significantly lessened than the joint Simon effect in the control group, demonstrating that when completing the joint action task in the state of acute stress, women’s ability to distinguishing self- from other-related mental representations was improved, and the strength of women’s action co-representation was diminished. The fNIRS results showed that when completing the joint Simon task in the state of the acute stress, the brain-to-brain synchrony at the r-TPJ in the stress group was significantly higher than that in the control group, demonstrating that the increased brain-to-brain synchrony at the TPJ may be served as the critical brain-to-brain neural mechanism underlying the joint action task under acute stress.

## 1. Introduction

The interpersonal interaction in the social context is one of the most important constitutional units in our daily lives. When completing the joint tasks with the specific common goal in the interactive scenario, people were required to coordinate with the co-actor by distinguishing different roles that were undertaken by all actors. During the joint task, the schema or the representation of our own and others’ actions and corresponding consequences was denoted as the action co-representation (Sebanz et al., 2006; van der Weiden et al., 2023). Our reaction could be automatically affected by the responses of the co-actor, and different contexts might also exert distinct impacts on our ability of self-other distinction in the action co-representation during the joint tasks (Sebanz et al., 2005; Dolk et al., 2014). Existing studies have adopted the joint Simon task to explore the psychological mechanism underlying the action co-representation (Dolk et al., 2014). When completing the joint Simon task, two participants in the dyad sitting side-by-side were, respectively, assigned to respond to distinct non-spatial stimulus characteristic regardless of the stimulus spatial position (Dolk et al., 2014). Despite the independence on the stimulus spatial position in the joint Simon task, the spatial incompatibility between the participant’s side and the stimulus position resulted in the decrease in the participant’s reaction time (RT) (Sebanz et al., 2003). The aforementioned phenomenon was denoted as the joint Simon effect (JSE). With the increase of the joint Simon effect, the strength of the action co-representation in the joint tasks was intensified, and the level of the difficulty for participants in self-other distinction was also boosted (Song et al., 2020). As the basic cognitive process in the joint task, the action co-representation during the joint tasks has played an essential role in the social interaction.

However, with the advent of VUCA (i.e., Volatility, Uncertainty, Complexity, and Ambiguity) era, the joint action in the interactive context under acute stress has been severely challenged. As one of the most indispensable procedures in the interpersonal interaction, the action co-representation may be altered by the unexpected stressors with the activation of the sympathetic-adrenal-medullary (SAM) and hypothalamic-pituitary-adrenal (HPA) axes (Von Dawans et al., 2012). Previous studies have provided preliminary behavioral evidence on how stress affected action co-representation. Research has reported that when participants jointly completed the task with the intimidating co-actor, the level of the shared task co-representation decreased, and the Joint Simon Effect disappeared (Hommel et al., 2009; Iani et al., 2011). It has also been verified that when participants completed the joint task in the competitive and threatening interaction context, participants, respectively, generated the self and other task representation, and the JSE also disappeared (Iani et al., 2014). One study using three task co-representation paradigms, respectively, examined the effect of acute stress on the shared representation on the perceptual, affective, and cognitive levels, demonstrating enhanced self-other distinction ability in the stressed women and decreased self-other distinction ability in the stressed men (Tomova et al., 2014). Even though the existing advance has revealed the preliminary evidence on the behavioral performance in the joint tasks under the state of the acute stress induced by different experimental paradigms, the underlying neural mechanism in the real-time interactive context still remained unknown due to the restricted neuroimaging techniques.

Recent advances regarding social interaction through a “second-person” approach have provided us with an innovative perspective on investigating the neural response pattern during the joint task under acute stress (Czeszumski et al., 2020). A growing number of studies using hyperscanning techniques uncovered cognitive and neural mechanism underlying the joint tasks (Liu and Pelowski, 2014). One study using a computer-based cooperation-competition game demonstrated increased inter-brain coupling in the superior frontal cortex in the cooperative condition (Cui et al., 2012). Another electroencephalography (EEG)-based hyperscanning study also verified brain-to-brain coupling in the cooperative condition using a computerized pong-game (Sinha et al., 2016). Recent advances further demonstrated the increased cooperative rate and enhanced theta/ alpha-band brain-to-brain synchronization in human-human condition than human-machine condition (Hu et al., 2018). Enhanced brain-to-brain correlation in inferior frontal cortex and inferior parietal lobe in the turn-based competition was also corroborated using the adapted pattern game paradigm (Liu et al., 2015).

Existing functional neuroimaging research has provided numerous evidence for the crucial involvement of the right temporal-parietal junction (r-TPJ) during the action co-representation. Previous research using the quantitative meta-analysis approach reported that the activation in the r-TPJ was involved in distinguishing between the self-initiated actions and the other-initiated actions by blocking the intrusive schemas and re-orienting the selective attention to either the self-reflection or the other-reflection (Murray et al., 2012). Relevant clinical evidence also reported that the schizophrenia patients with the lesion or dysfunction in the TPJ exhibited no Joint Simon Effect (JSE), indicating the deficits in the self-other integration in the schizophrenia patients (Liepelt et al., 2012). The latest advance adopting the hyperscanning technique reported that the brain-to-brain synchrony in bilateral TPJ was recruited in the action co-representation in both competitive and cooperative contexts, and the brain-to-brain synchrony was significantly associated with the joint action performance (Yang et al., 2021). It was also found that under the unpredictable threatening circumstances, the enhanced brain-to-brain synchrony at the r-TPJ was also observed in the cooperative interactive tasks (Zhao et al., 2022; Yin et al., 2023). Hence, the r-TPJ was selected as the region of interest (ROI) in the current study to explore the behavioral performance and the brain-to-brain synchrony underlying the joint action task under acute stress with the fNIRS-based hyperscanning method.

Taken together, the current study intended to provide the comprehensive understanding of the brain-to-brain synchrony pattern underlying the real-time action co-representation in the state of the acute stress with the application of the fNIRS-based hyperscanning method in the “second-person” approach. On the basis of the existing research, the current study hypothesized that the state of acute stress would boost women’s self-other distinction ability, and diminish the strength of women’s action co-representation in the joint Simon task (Tomova et al., 2014). Corresponding brain-to-brain synchrony at the r-TPJ was also expected in the current study.

## 2. Methods

To avoid the possible confusion caused by gender differences under acute stress, 80 female undergraduate students from Shaanxi Normal University (age: 20.25 ± 1.20 years) composing 40 dyads were recruited. All participants were prescreened by the exclusionary criteria that aligned with prior studies (Zhao et al., 2021). All dyads were randomly allocated to the stress group (*N* = 20) and the control group (*N* = 20). Prior to the formal experiment, all participants signed the written informed consent. The present study abided by the Declaration of Helsinki, and was ratified by Academic Committee of Key Laboratory of Modern Teaching Technology, Ministry of Education, Shaanxi Normal University in China.

### 2.1. Experimental procedures

**Figure 1:**
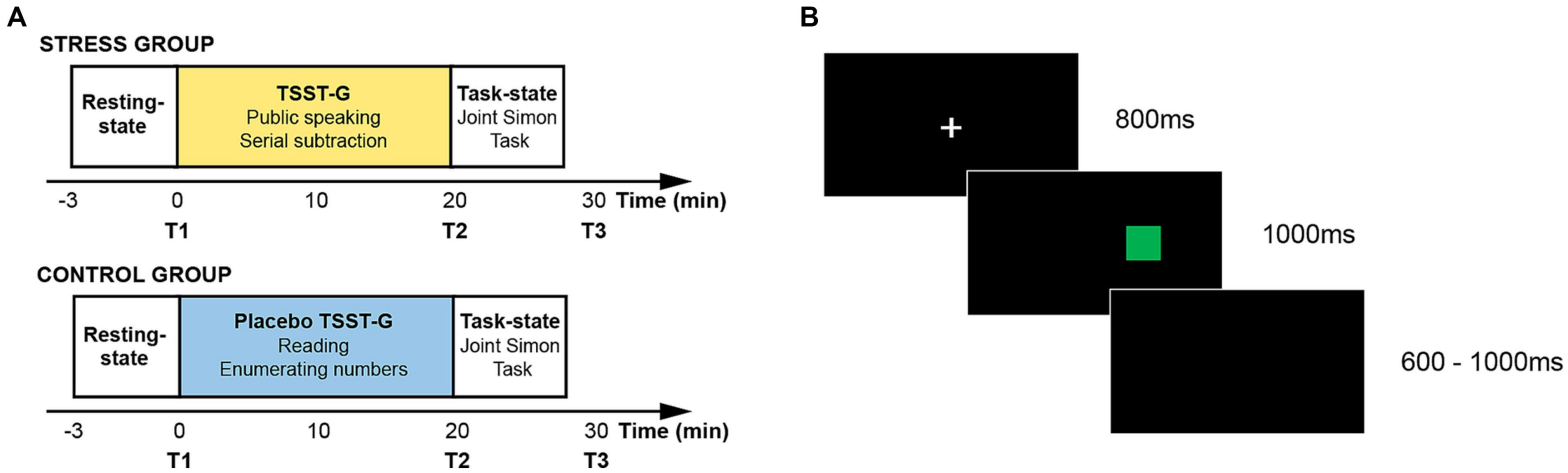
**(A)** Experimental timeline; **(B)** Experimental flow in one trial.

All experiments were carried out between 14:00 and 18:00 to avoid daily fluctuation of cortisol levels (Izawa et al., 2010). The formal experimental procedure was composed of three sessions, i.e., a resting-state session, a stress (or placebo) induction session, and a task-state session. To verify whether the acute stress state was successfully induced, the present study collected physiological indicators including salivary cortisol and heart rate which, respectively, represented the activation of the HPA axis and the SAM axis (Duan et al., 2019; Kan et al., 2019; Wang et al., 2019; Duan et al., 2020a). The salivary samples were collected after the resting-state session (T1), the stress (or placebo) induction session (T2), and the task-state session (T3). The experimental time flow has been presented in Figure 1A.

During the resting-state session, participants were asked to rest with their eyes closed for 3 min. The brain-to-brain synchrony during the resting-state session was adopted as the baseline in the subsequent fNIRS data analysis. During the stress (or placebo) induction session, the dyads in the stress group and the control group, respectively, performed the Trier Social Stress Test for Groups (TSST-G) and the placebo TSST-G. TSST-G has been regarded as a standard experimental paradigm to induce the acute stress state in the form of the group, and has been extensively adopted in exploring the group behavior under acute stress (Von Dawans et al., 2011). When performing the TSST-G session, the dyads in the stress group were required to complete two sub-tasks. The first sub-task required the dyads to make a five-minute speech to introduce themselves by turns in a mock job interview. The interview panel was composed of two trained experimenters in the white gowns. The second sub-task required the dyads to serially subtract a fixed double-digit number from a given four-digit number for 5 min by turns. The whole TSST-G session was recorded by the digital videos in front of the dyads. When performing the placebo TSST-G session, the dyads in the control group were also required to complete two sub-tasks which were devised in the identical manner without the exposure to the interview panel and the digital videos. The first sub-task required the dyads to read the given material in undertones for 5 min. The second sub-task required the dyads to enumerate the numbers from a given number in undertones for 5 min.

During the task session, the present study adopted the Joint Simon Task to assess the dyads’ action co-representation under acute stress. Two participants in a dyad sat side by side to jointly complete the task. The participant on the left side was designated as Actor A, and the participant on the right side was designated as Actor B. The Joint Simon Task was programmed and presented by E-prime 2.0. Aligning with the standard Simon task and prior studies, the present study adopted the red and green squares as the task stimuli (Yang et al., 2021). The experimental timeline of one trial has been illustrated in Figure 1B. The white fixation cross was firstly presented on the black background for 800 ms. The red and green squares were then randomly presented on either the left side or the right side. Participants were required to respond to the stimulus color regardless of its spatial location within 1,000 ms. Actor A was required to respond to the red squares by pressing the “Z” key on the keyboard, and Actor B was required to respond to the green squares by pressing the “/” key on the keyboard. The accuracy rate and reaction time were recorded. The formal experiment was composed of 128 trials in two blocks, and the color and the location of the stimulus were counterbalanced. The inter-trial intervals varied from 600 ms to 1,000 ms at random.

### 2.2. Data collection

#### 2.2.1. Acute-stress-related-data collection

Aligning with the existing studies, the present study collected physiological indicators including the heart rate and salivary cortisol which, respectively, represented the activation of the SAM and the HPA to validate whether the acute stress state was effectively induced (Rosenbaum et al., 2018).

Regarding the recording of the heart rate, the present study consecutively recorded participants’ heart rate using the BIOPAC MP150 amplifier system with Ag/AgCl electrodes during the entire experiment. The sampling rate was set as 1,000 Hz.

Regarding the collection of the salivary samples, the present study collected participants’ salivary samples using the Salivette^®^ (SARSTEDT, Product Number: 51.1534.500, Germany). Participants’ salivary samples were, respectively, collected after the resting-state session (T1), the stress (or placebo) induction session (T2), and the task-state session (T3).

#### 2.2.2. fNIRS data acquisition

The current study used a 38-channel LABNIRS equipment to synchronously collect the dyads’ fNIRS signals. The sampling rate of the fNIRS data was 10 Hz. Aligning with prior studies, the current study was merely centered on HbO time series due to the high signal-to-noise rate (Cui et al., 2012). Due to the involvement in interpersonal interaction, the region of interest (ROI) covered the inferior parietal lobule (IPL), angular gyrus (AG), superior temporal gyrus (STG), and inferior frontal gyrus (IFG) (Liu et al., 2015).

**Figure 2:**
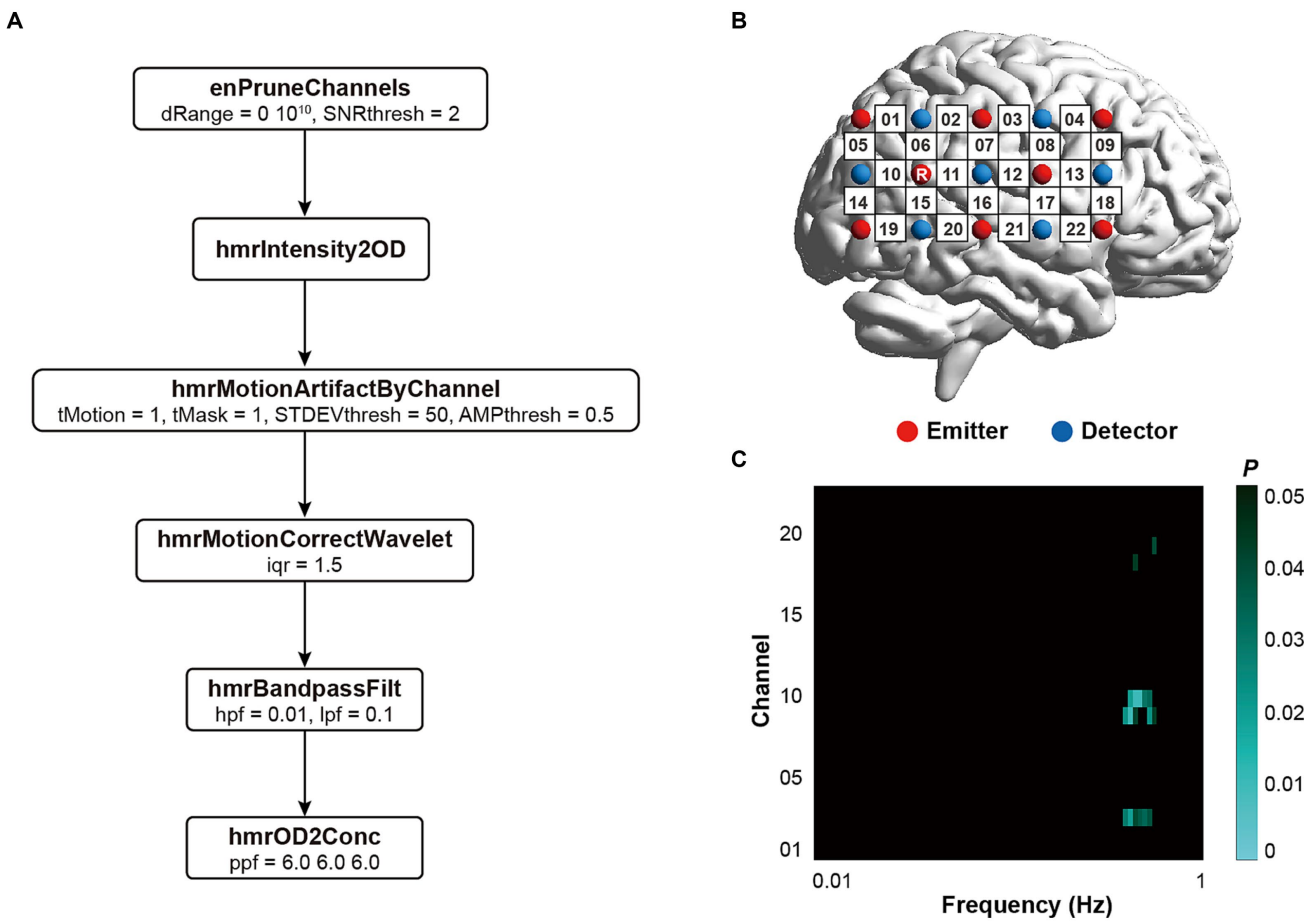
fNIRS data analysis. **(A)** Preprocessing pipeline; **(B)** Positioning of the optical probe patch; **(C)** Frequency band of interest.

As illustrated in Figure 2, a 3 × 5 patch with 15 optical probes (8 emitters and 7 detectors) was put on the region of interest with 30 mm distance. The positioning of the patch aligned with the standard international 10–20 system. The bottom line of the optical probe patch aligned at the sagittal reference curve, and the P6 was set as the referential optode. The current study adopted the FASTRAK space digitizer to locate the anatomical positions of optodes and channels (CHs), and the spatial coordinates were further calculated by the NIRS_SPM MATLAB package (Ye et al., 2009). The location information of all channels in the ROI was illustrated in the Supplementary material. The visualization of ROI was drawn by the BrainNet Viewer MATLAB package (Xia et al., 2013).

### 2.3. Data analysis

#### 2.3.1. Acute-stress-related-data analysis

Regarding the salivary cortisol, the present study adopted the enzyme-linked immunosorbent assay (ELISA, ZCIBIO Technology Co., Ltd., China) to determine the salivary cortisol concentration in the collected salivary samples which were promptly frozen at −20°C after the collection at the resting-state session (T1), the stress (or placebo) induction session (T2), and the task-state session (T3).

Regarding the heart rate, the present study adopted AcqKnowledge 5.0 software to calculate the averaged heart rate during the resting-state session, the stress (or placebo) induction session, and the task-state session. Beats per minute (BPM) were adopted to describe the heart rate in the present study.

#### 2.3.2. Behavioral data analysis

With the reference to the prior studies, the accuracy rate (%) and the reaction time (RT) for the correct responses were adopted in the behavioral data analysis (Yang et al., 2021). The trials with the reaction time that exceeded ±2.5 SDs were precluded in the following analysis. We calculated the indicator of the Joint Simon Effect (JSE) by subtracting the reaction time of the compatible trials from the reaction time of the incompatible trials (Yang et al., 2021). The JSE was defined as follows. The independent-sample *t*-tests were, respectively, conducted on the accuracy rate and the JSE.


$$\mathrm{JSE}={\mathrm{RT}}_{\mathrm{Incmpatible}}-{\mathrm{RT}}_{\mathrm{Compatible}}$$

#### 2.3.3. fNIRS data analysis

The fNIRS data in the current study were preprocessed by HOMER2 MATLAB package. The quality of all fNIRS signals were firstly inspected by the enPruneChannels function. The channels with poor signals were replaced with the mean fNIRS signals of the most adjacent channels. The raw fNIRS signals were transformed into the optical density by the hmrIntensity2OD function. The motion artifact in the signals was detected and further corrected by the hmrMotionArtifactByChannel function and the hmrMotionCorrectWavelet function. The hmrBandpassFilt was further applied on the signals to exclude the noise with low and high frequencies. The optical density signals were finally transformed into the concentration by the hmrOD2Conc function. The whole processing pipeline and the corresponding parameters have been illustrated in Figure 2.

The brain-to-brain synchrony, i.e., inter-brain synchronization (IBS), was further calculated by the wavelet transform coherence (WTC) MATLAB package (Grinsted et al., 2004). Aiming at precisely ascertaining the frequency of interest (FOI) in the whole calculation, we used a set of paired-sample *t*-tests with False Discovery Rate (FDR) correction to check whether the brain-to-brain synchrony during the task-state session was significantly different from the brain-to-brain synchrony during the resting-state session across the whole frequency range, i.e., 0.01 Hz-1 Hz (Pan et al., 2020; Zhao et al., 2023). The brain-to-brain synchrony was averaged across all times and all channels at each frequency band within each dyad. We detected that the brain-to-brain synchrony in the task-state session was higher than the brain-to-brain synchrony in the resting-state session in the frequency band from 0.403 Hz to 0.571 Hz which was chosen as the FOI in the current study. The chosen FOI excluded the fluctuations with extremely low frequencies (i.e., below 0.1 Hz) and the possible physiological noises covering the Mayer waves (0.1 Hz), respiration (0.2–0.3 Hz), and cardiac pulsation (0.7–4 Hz) (Nozawa et al., 2016).

The brain-to-brain synchrony during the resting-state session and the brain-to-brain synchrony during the task-state session in the chosen FOI was further, respectively, averaged across all dyads and all channels. Aligning with the prior studies, the current study further analyzed the task-related brain-to-brain synchrony by subtracting the brain-to-brain synchrony during the resting-state session from the brain-to-brain synchrony during the task-state session, and the Fisher *z* transformation was conducted on the calculated task-related brain-to-brain synchrony (Duan et al., 2020b). A series of independent-sample *t*-tests with False Discovery Rate (FDR) correction were then adopted to compare the task-related brain-to-brain synchrony in the stress group with the task-related brain-to-brain synchrony in the control group.

## 3. Results

### 3.1. Acute-stress-related indicators

#### 3.1.1. HPA axis indicator: salivary cortisol levels

**Figure 3:**
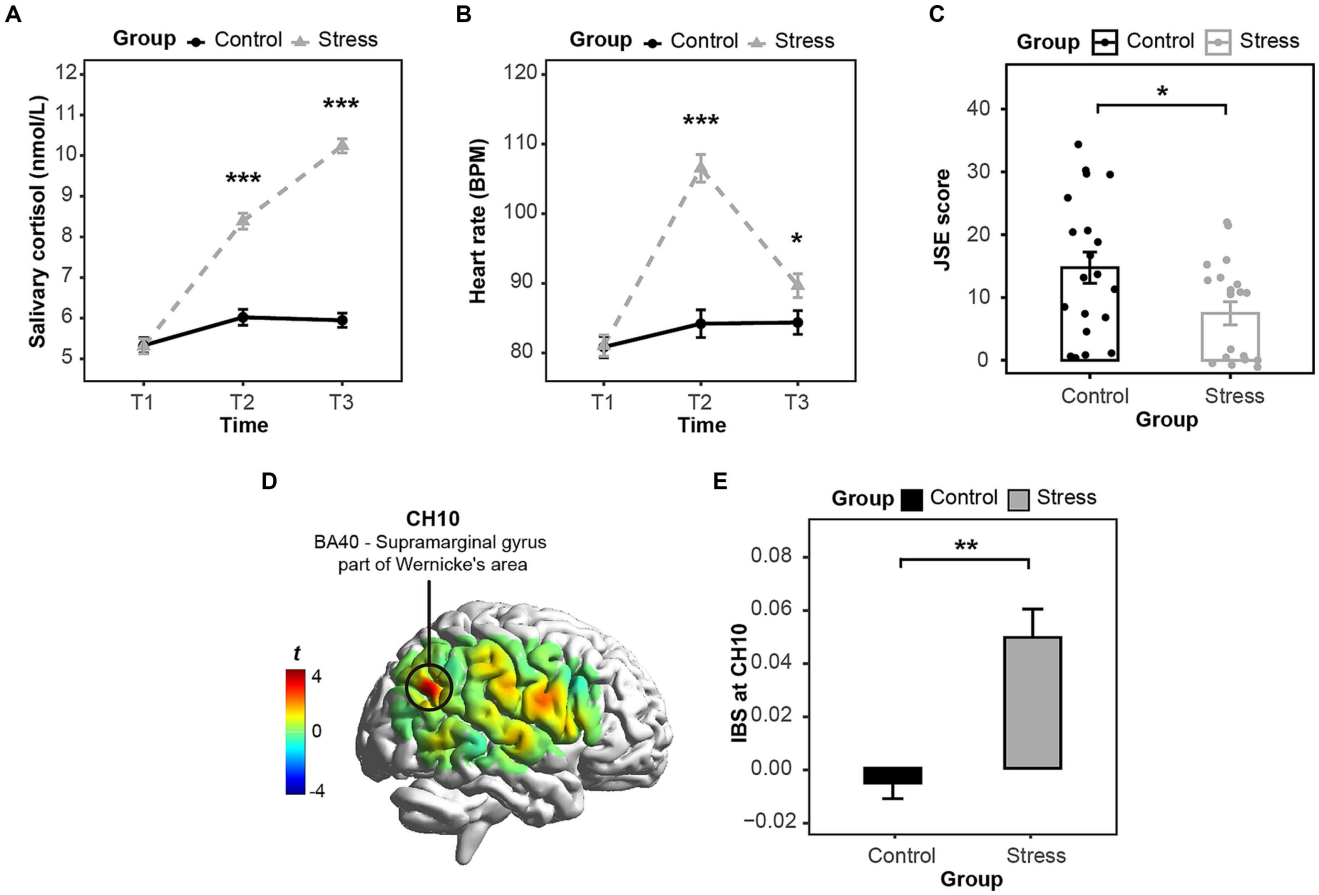
**(A)** The variation of salivary cortisol levels in two groups; **(B)** The variation of heart rates in two groups; **(C)** The Joint Simon Effect scores in two groups; **(D)** The t-maps of the task-related IBS; **(E)** The averaged task-related IBS at CH10 in two groups. *p < 0.05, **p < 0.01, ***p < 0.001.

A repeated-measures ANOVA with GROUP as a between-participant factor and TIME as a within-participant factor was implemented on the salivary cortisol levels in the collected saliva samples. The current study found the significant main effects of TIME, *F* (2, 156) = 139.37, *p* < 0.001, *η_p_*^2^ = 0.64, and GROUP, *F* (1, 78) = 159.84, *p* < 0.001, *η_p_*^2^ = 0.67. Our results also manifested the significant interaction effect of TIME × GROUP, *F* (2, 156) = 81.03, *p* < 0.001, *η_p_*^2^ = 0.51. The simple effect analysis reported that the salivary cortisol levels of the stress group were significantly higher than the salivary cortisol levels of the control group at T2 (*p* < 0.001) and T3 (*p* < 0.001) (see Figure 3A). Our results corroborated that the acute stress state was successfully activated on the HPA axis in the current study.

#### 3.1.2. SAM axis indicator: averaged heart rate

A repeated-measures ANOVA with GROUP as a between-participant factor and TIME as a within-participant factor was implemented on the averaged heart rate in three sessions. The current study found the significant main effects of TIME, *F* (2, 156) = 136.42, *p* < 0.001, *η_p_*^2^ = 0.64, and GROUP, *F* (1, 78) = 16.85, *p* < 0.001, *η_p_*^2^ = 0.18. Our results also manifested the significant interaction effect of TIME × GROUP, *F* (2, 156) = 87.48, *p* < 0.001, *η_p_^2^* = 0.53. The simple effect analysis reported that the averaged heart rate of the stress group was significantly higher than the averaged heart rate of the control group at T2 (*p* < 0.001) and T3 (*p* < 0.05) (see Figure 3B). Our results further corroborated that the acute stress state was successfully activated on the SAM axis, and the manipulation of the acute stress induction in the current study was effective.

### 3.2. Behavioral performance in the joint Simon task

The independent-sample *t*-tests were, respectively, implemented on the accuracy rate and the JSE in the Joint Simon task. Our results on the accuracy rate reported that no significant difference was found between the accuracy rate in the stress group and the control group, *t* (38) = 0.38, *p* = 0.71, Cohen’s *d* = 0.12. The averaged accuracy rates of the stress group (99.26% ± 0.86%) and the control group (99.14% ± 1.07%) both exceeded 99%, demonstrating that participants have fully understood the task requirements. Our results on the JSE reported that the JSE of the stress group was significantly lower than the JSE of the control group, *t* (38) = −2.348, *p* < 0.05, Cohen’s *d* = 0.74 (see Figure 3C). The results above corroborated that acute stress improved the actor’s ability to distinguishing self- from other-related mental representations on the behavioral level.

### 3.3. Brain-to-brain synchrony during the joint Simon task

A series of independent-sample *t*-tests with FDR correction were adopted to compare the task-related brain-to-brain synchrony in the stress group with the task-related brain-to-brain synchrony in the control group. Our results only reported the significant results in CH10, i.e., the right inferior parietal cortex (r-IPL, Brodmann Area 40) (see Figure 3D). The brain-to-brain synchrony at CH10 in the stress group was significantly higher than the brain-to-brain synchrony at CH10 in the control group, *t* (38) = 3.451, *p* < 0.01, Cohen’s *d* = 1.09 (see Figure 3E). Our results on the brain-to-brain synchrony during the Joint Simon Task corroborated that the brain-to-brain synchrony at the r-IPL in the stress group was higher than the brain-to-brain synchrony in the control group.

### 3.4. The IBS-behavioral correlation

The current study further, respectively, examined the association between the dyads’ behavioral performance and the brain-to-brain synchrony at CH10 in both the stress group and the control group. The Pearson correlation analysis was implemented on the aforementioned results. Our results on the IBS-behavioral correlation reported that no significant correlation between the JSE score and the brain-to-brain synchrony at CH10 was found in both the stress group (*r* = −0.106, *p* = 0.658) and the control group (*r* = −0.424, *p* = 0.062).

## 4. Discussion

The current study examined the behavioral performance and the corresponding inter-brain mechanism during the Joint Simon Task under acute stress with the fNIRS-based hyperscanning method. On the basis of the existing evidence, the current study made the first dedication on unraveling the unique brain-to-brain synchrony pattern underlying the real-time action co-representation in the state of the acute stress with the “second-person” approach. The discussion on the results in the current study were, respectively, delineated from the physiological—psychological—neural perspective as follows.

Firstly, the current study examined whether the acute stress state was successfully induced by the TSST-G experimental paradigm on the physiological level using the averaged heart rate as the SAM axis indicator, and the salivary cortisol levels as the HPA axis indicator. Our results showed that the salivary cortisol levels and the heart rate of the stress group were significantly higher than that of the control group after the stress (or placebo) induction session and the task-state session. Aligning with prior studies, our results on the physiological level re-corroborated that the TSST-G experimental paradigm could effectively activate participants’ acute stress state on both the HPA axis and the SAM axis (Von Dawans et al., 2011; Zhao et al., 2022).

Secondly, the current study investigated the effect of the acute stress state on the real-time action co-representation on the behavioral level adopting the JSE scores as the indicator of the self-other distinction level in the Joint Simon Task. Our results reported that when comparing with the dyads in the control group, the stressed dyads that experienced the acute stress elicited by the TSST-G experimental paradigm acquired lower JSE scores in the subsequent joint Simon task. The JSE has been attributed to the shared co-representation of the whole task-set in a dedicated and automatic manner (Yamaguchi et al., 2018). It was found that the JSE could be influenced by the interpersonal relationships (Hommel et al., 2009), interactive modes (Iani et al., 2011; Yang et al., 2021), and emotional context (Kuhbandner et al., 2010). Supporting the proposed hypothesis, our behavioral results justified that the state of acute stress boosted women’s self-other distinction ability, and diminished the strength of women’s action co-representation in the joint Simon task which were also consistent with the existing evidence (Tomova et al., 2014).

Thirdly, the current study further explored the corresponding brain-to-brain synchrony pattern underlying the real-time action co-representation in the state of the acute stress on the neural level with the application of the fNIRS-based hyperscanning method in the “second-person” approach. Supporting the proposed hypothesis, our results demonstrated that when completing the joint Simon task in the state of the acute stress, the brain-to-brain synchrony at the r-TPJ (BA40) in the stress group was significantly higher than that in the control group. Existing neuroimaging evidence has shown that the r-TPJ played an indispensable role during the self-other distinction (Aichhorn et al., 2006; Lawrence et al., 2006; Hoffmann et al., 2016), and following studies using transcranial direct current stimulation (tDCS) also further provided strong evidence that r-TPJ was causally recruited in self and other representation (Santiesteban et al., 2012). Recent literature with the second-person approach also demonstrated that enhanced inter-brain synchrony at the r-TPJ was correlated with the trust and shared intentionality (Tang et al., 2016; Cheng et al., 2022). On the basis of the existing studies, the current study further corroborated that when completing the joint action task in the state of the acute stress, the increased inter-brain connectivity at the r-TPJ (BA40) which manifested the increased level of the shared intentionality may be served as the critical brain-to-brain neural mechanism underlying the joint action task under acute stress.

Several limitations of the present study were listed as follows. First of all, we only recruited the female participants to avoid the reported effect of gender composition the brain-to-brain synchrony (Lu et al., 2020). Further research is also expected to examine whether different gender composition would affect turn-based interaction under acute stress. In addition, we only detected fNIRS data at the ROI region due to the limitation of the equipment. Future research is expected to explore the brain-to-brain synchrony at the whole brain with more advanced neuroimaging means.

## 5. Conclusion

With the application of the fNIRS-based hyperscanning method in the “second-person” approach, the current study examined the brain-to-brain synchrony pattern underlying the real-time action co-representation in the state of the acute stress. Our behavioral results showed that the joint Simon effect was found in both the stress group and the control group, but the joint Simon effect in the stress group was significantly lower than that in the control group, corroborating that when completing the joint action task in the state of acute stress, women’s ability to distinguishing self- from other-related mental representations was improved, and the strength of women’s action co-representation was diminished. Our fNIRS results showed that when completing the joint Simon task in the state of the acute stress, the brain-to-brain synchrony at the r-TPJ in the stress group was significantly higher than that in the control group, demonstrating that the increased brain-to-brain synchrony at the TPJ may be served as the critical brain-to-brain neural mechanism underlying the joint action task under acute stress.

## Data availability statement

The raw data supporting the conclusions of this article will be made available by the authors, without undue reservation.

## Ethics statement

The studies involving humans were approved by Academic Committee of Key Laboratory of Modern Teaching Technology, Ministry of Education, Shaanxi Normal University in China. The studies were conducted in accordance with the local legislation and institutional requirements. The participants provided their written informed consent to participate in this study.

## Author contributions

SL: conceptualization and writing. HZ: data curation and data analysis. HD: conceptualization, funding acquisition, resources, and supervision. All authors contributed to the article and approved the submitted version.

## Funding

This study was supported by the National Natural Science Foundation of China Grant (32071078), the Fundamental Research Funds for the Central Universities (GK202201016), the Research Program Fund for the Youth Innovation Team of Shaanxi Universities, the Research Program Fund of the Collaborative Innovation Center of Assessment toward Basic Education Quality at Beijing Normal University (2022-05-009-BZPK01), the Research Project of Teacher Education Reform and Teacher Development of Shaanxi Province (SJS2022YB013).

## Conflict of interest

The authors declare that the research was conducted in the absence of any commercial or financial relationships that could be construed as a potential conflict of interest.

## Publisher’s note

All claims expressed in this article are solely those of the authors and do not necessarily represent those of their affiliated organizations, or those of the publisher, the editors and the reviewers. Any product that may be evaluated in this article, or claim that may be made by its manufacturer, is not guaranteed or endorsed by the publisher.
