# Supplementary material for: Brain-to-brain synchrony during dyadic action co-representation under acute stress: evidence from fNIRS-based hyperscanning
Source: Front Psychol. 2023 Sep 6;14:1251533. doi: 10.3389/fpsyg.2023.1251533 (PMC10511757; doi:10.3389/fpsyg.2023.1251533)
Supplement: Supplementary file 1 [file Table_1.DOCX]

Supplementary Material

# Location information of all channels

The location information of all channels in the region of interest (ROI) was illustrated as follows.

**Table S1 Location information of all channels**

| CH | Brodmann Area (Percentage of Overlap) | MNI | | |
| --- | --- | --- | --- | --- |
|  |  | X | Y | Z |
| 1 | BA40 - Supramarginal Gyrus (95%) | 53 | -56 | 54 |
| 2 | BA40 - Supramarginal Gyrus (85%) | 61 | -36 | 54 |
| 3 | BA3 - Primary Somatosensory Cortex (39%) | 59 | -17 | 54 |
| 4 | BA6 - Pre-Motor and Supplementary Motor Cortex (71%) | 52 | 8 | 51 |
| 5 | BA39 - Angular Gyrus (59%) | 53 | -68 | 43 |
| 6 | BA40 - Supramarginal Gyrus (100%) | 64 | -42 | 47 |
| 7 | BA2 - Primary Somatosensory Cortex (29%) | 67 | -25 | 45 |
| 8 | BA6 - Pre-Motor and Supplementary Motor Cortex (96%) | 62 | -1 | 42 |
| 9 | BA9 - Dorsolateral Prefrontal Cortex (65%) | 52 | 25 | 41 |
| 10 | BA40 - Supramarginal Gyrus (93%) | 64 | -54 | 34 |
| 11 | BA40 - Supramarginal Gyrus (72%) | 70 | -30 | 36 |
| 12 | BA6 - Pre-Motor and Supplementary Motor Cortex (72%) | 68 | -8 | 33 |
| 13 | BA9 - Dorsolateral Prefrontal Cortex (68%) | 63 | 13 | 29 |
| 14 | BA39 - Angular Gyrus (73%) | 60 | -67 | 19 |
| 15 | BA40 - Supramarginal Gyrus (45%) | 71 | -38 | 21 |
| 16 | BA43 - Subcentral Area (41%) | 70 | -15 | 23 |
| 17 | BA6 - Pre-Motor and Supplementary Motor Cortex (57%) | 67 | 5 | 23 |
| 18 | BA46 - Dorsolateral Prefrontal Cortex (52%) | 59 | 31 | 17 |
| 19 | BA22 - Superior Temporal Gyrus (52%) | 71 | -48 | 5 |
| 20 | BA22 - Superior Temporal Gyrus (46%) | 73 | -24 | 6 |
| 21 | BA22 - Superior Temporal Gyrus (51%) | 68 | -4 | 9 |
| 22 | BA45 - Pars Triangularis (73%) | 61 | 25 | 10 |
